# Spraying Respiratory Epithelial Cells to Coat Tissue-Engineered Constructs

**DOI:** 10.1089/biores.2015.0015

**Published:** 2015-06-01

**Authors:** Anja Lena Thiebes, Stefanie Albers, Christian Klopsch, Stefan Jockenhoevel, Christian G. Cornelissen

**Affiliations:** ^1^ Department of Tissue Engineering and Textile Implants, Institute of Applied Medical Engineering, Helmholtz Institute, RWTH Aachen University, Aachen, Germany.; ^2^ Clinic and Policlinic for Cardiac Surgery, University of Rostock, Rostock, Germany.; ^3^ Aachen-Maastricht Institute for Biobased Materials, Maastricht University at Chemelot Campus, Geleen, The Netherlands.; ^4^ Section for Pneumology, Department for Internal Medicine, Medical Faculty, RWTH Aachen University, Aachen, Germany.

**Keywords:** aerosolization, cell seeding, cell transfer, respiratory epithelium, tissue engineering, viability

## Abstract

Applying cells in a spray can overcome current hurdles in coating tissue engineered constructs with a thin layer of endo- or epithelial cells. We report here a structured study on the influences of spray application with a medical spray device on vascular smooth muscle cells (vSMCs) and respiratory epithelial cells (RECs) with and without fibrin gel. Next to viability and cytotoxicity assays, the *in vitro* differentiation capacity after spray processing was analyzed. For vSMC, no influence of air pressures till 0.8 bar could be shown, whereas the viability decreased for higher pressures. The viability of RECs was reduced to 88.5% with 0.4 bar air pressure. Lactate dehydrogenase-levels in the culture medium increased the first day after spraying but normalized afterward. In the short term, no differences by means of morphology and expression-specific markers for vSMCs and RECs were seen between the control and study group. In addition, in a long-term study for 28 days with the air–liquid interface, RECs differentiated and built up an organized epithelial layer with ciliary development that was comparable to the control for cells sprayed without fibrin gel. When spraying within fibrin gel, ciliary development was lower at 28 days. Thus, spraying of vSMCs and RECs was proved to be a suitable method for tissue engineering. Especially for RECs, this application is of special significance when coating luminal structures or other unfavorable topographies.

## Introduction

The technique of spraying cells was proposed by Bahoric et al. with an *in vitro* study in which epidermal cells were sprayed on cell culture plates with a pump-action aerosol nozzle.^1^ Since then, researchers have accomplished both *in vivo* and *in vitro* studies with different experimental setups and techniques. Various types of spraying devices were used: commercially available airbrush pistols,^2–4^ pump heads,^1,5^ atomizers,^6^ and clinically used spray nozzles.^7–9^ However, systematic studies on the influence of spray processing on different cell types are still sparse. Especially for the field of tissue engineering, spraying is a highly promising option: As necessary for epithelial or endothelial cells, thin (single) cell layers can be applied onto tissue-engineered constructs.

*In vitro* studies have revealed diverse results on cell behavior and survival after spraying depending on procedure and cell type. When analyzing the influence of nozzle diameters and air pressures on fibroblast survival, decreasing viability with increasing pressures and decreasing airbrush nozzle diameter with viabilities ranging between 37% and 94% was found.^4^ As described by Duncan et al., different hydrostatic, shear, and elongation stresses act on the cells and might be a reason for this variance.^10^

Looking at *in vivo* experiments and clinical studies, different models were investigated. So far, the main focus was on wound healing for burn or chronic wounds,^5,7,11–13^ Better epithelial coverage and histological results in a porcine wound closure model have been shown when spraying epithelial cells.^11^ Similar results have been obtained by Goedkoop et al., who have clinically tested the effect of keratinocytes and fibroblasts delivered in a fibrin matrix on closure of chronic leg ulcers.^5^

Fibrin has been frequently used when spraying cells onto different surfaces^7–9,13–15^: Hafez et al. proved the positive impact of spraying a mixture of smooth muscle cells and urothelial cells with fibrin gel on decellularized colonic segments for bladder augmentation in piglets.^12^ Fibrin is clinically used as tissue glue in plastic surgery and also applied for various tissue engineering applications, as it can be isolated autologously.^16,17^ For spraying of cells, fibrin has the advantage that it polymerizes fast to keep the cells in place but degradation takes place within a few days, which then allows (epithelial) cells to differentiate *in situ*. Fibrin gel is, hence, a very suitable material for this approach.

Although only little research on cell spraying has been carried out for tissue engineering of the respiratory tract, we propose that it is a viable option to seed epithelial cells on the luminal surface of tissue-engineered tubular constructs. So far, only one study analyzed growth kinetics of tracheal epithelial cell when sprayed with an atomizer on microscope slides and no significant differences to pipetted controls were found.^6^

In our lab, endothelial cells are successfully coated on a viable tissue-engineered construct based on a fibrin gel scaffold as shown by Weinandy et al. with the BioStent concept.^18^ The same approach with respiratory epithelial cells (RECs) was not successful (upublished data). However, the suitability of fibrin gel for culture and differentiation of RECs was already shown in our lab.^19^

Thus, to directly apply the cells where desired, spraying is highly interesting. The main objectives for this study are to assess the general suitability of a commercially available spray device (Tisseel, EASYSPRAY set; Baxter) and to find the best spraying conditions as determined by ovine vascular smooth muscle cell (vSMC) and REC survival, proliferation, and lysis. Furthermore, differentiation of both cell types will be proved, especially the long-term differentiation of the RECs with ciliary development after spraying with and without fibrin gel.

## Materials and Methods

### Cell culture

Ovine carotid arteries (with a length of 10 cm) and tracheas (with a length of 5–7 cm) were harvested from sheep euthanized for other purposes at the Institute of Laboratory Animal Science in the University Hospital Aachen under sterile conditions and immediately placed in transport buffer (100 mM HEPES, 140 mM NaCl [Sigma-Aldrich^®^], 2.5 mM KCl, 10 mM glucose [both Merck], 1% antibiotic-antimycotic solution [ABM; Gibco^®^]; pH 7.4). The procedures used conformed to the “Guide for the care and use of laboratory animals” published by the US National Institutes of Health (The National Academies Press, 2011).

A mixed population of smooth muscle cells/fibroblasts (vSMC) was harvested by outgrowth from ovine carotid artery rings in Dulbecco's modified essential medium (DMEM; Gibco) supplemented with 10% fetal bovine serum (Gibco) and 1% ABM as described by Tschoeke et al.^20^ RECs were isolated according to a protocol first published by Yamaya et al.^21^: The mucosa was incised longitudinally; mucosa strips were removed, placed in a solution of 1.8 U/mL protease XIV (Sigma-Aldrich), and incubated at 4°C overnight. After removal of the strips and centrifugation, the cells (∼5×10^5^) were dispersed in DMEM, seeded in cell culture flasks, and maintained in a humidified incubator at 37°C and 5% CO_2_. After 24 h, the medium was changed to Airway Epithelial Cell Growth Medium (PromoCell). When cells reached 70–80% of confluence, cells were detached using 0.05% trypsin/0.02% EDTA solution (PAN-Biotech). RECs in passage 1 (after 2 weeks of culture) and vSMCs till passage 5 (∼6–8 weeks in culture) were used for this study.

### Spraying experiments

**FIG. 1.**
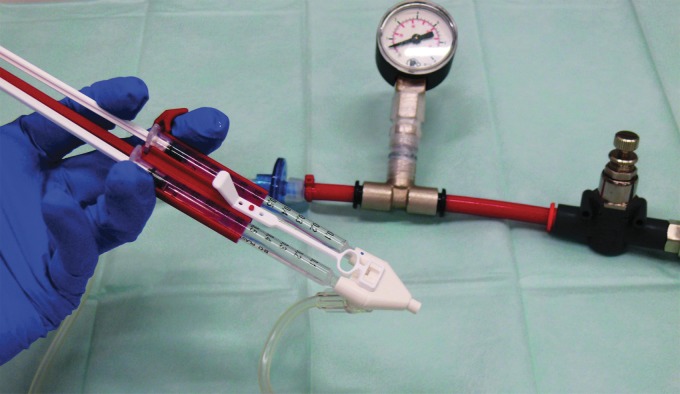
Spray setup with pressure regulator and -gauge (at the top), spraying nozzle (front), and syringe holder with syringes (left).

For an overview of all experiments, please see Table 1. The spraying setup is shown in Figure 1. All cell experiments were accomplished under sterile conditions. For the spraying experiments, Tisseel EASYSPRAY Sets™ (Baxter) were used with two 1 mL syringes (BD Plastipak™). For experiments with fibrin gel, commercial fibrinogen (20 mg/mL; Calbiochem^®^) and polymerization solution (Tris-buffered CaCl_2_ [7.5 mM] and 6 IU/mL thrombin) were prepared as previously described and cells were resuspended in the polymerization solution.^22^ Fibrinogen and polymerization solution were drawn into separate syringes. For all other experiments, the cell suspension was applied with both syringes simultaneously. Distance to the substrate was 2 cm. Air pressure was adjusted to values between 0.4 and 2 bar with a pressure gauge. In the first experiment, the pressure was varied to analyze its influence on vSMC survival; cell suspension was injected by hand with a flow rate of 7–10 mL/min. For all following experiments, the pressure was set to 0.4 bar. In the second experiment, the cell suspension was injected with a syringe pump (Perfusor^®^ Compact; B. Braun) with the indicated flow velocities of 10, 30, 55, 75, and 95 mL/min. Further, the suspension was injected by hand as fast as possible (120–150 mL/min), named “manual” in the diagram.

**Table 1.** **Overview of Experiments Conducted in This Study for the Respective Cell Type**

Vascular smooth muscle cells	Respiratory epithelial cells
Viability for different pressures (0, 0.4, 0.8, 1.2, 1.6, 2 bar)	Viability (0.4 bar)
Viability for different velocities (10, 30, 55, 75, 95 mL/min)	Short-term differentiation (pan-cytokeratin expression)
Short-term differentiation (α-SMA expression)	Long-term differentiation (14 and 28 days: pan-cytokeratin expression and PAS reaction)

α-SMA, α-smooth muscle actin; PAS, periodic acid Schiff's reaction.

For all short-term experiments, a 200 μL cell suspension was added to wells of a 12-well plate (CellStar^®^; Greiner Bio-One) with a density of 5×10^4^ and 8×10^4^ cells/cm^2^ for vSMCs and RECs, respectively. As a positive control, the same setup was used without air flow. For all statistically analyzed experiments, the number of samples was 5; from each sample, five images were taken at distinct areas of the well. For the cell survival and differentiation studies, cells were resuspended in Dulbecco's phosphate-buffered saline (PBS; Gibco) or in the respective culture medium. For differentiation analysis, cells were cultured for 3–5 days until confluency and then fixed and stained as described next. Visual inspection of cell morphology and medium change were done daily.

**FIG. 2.**
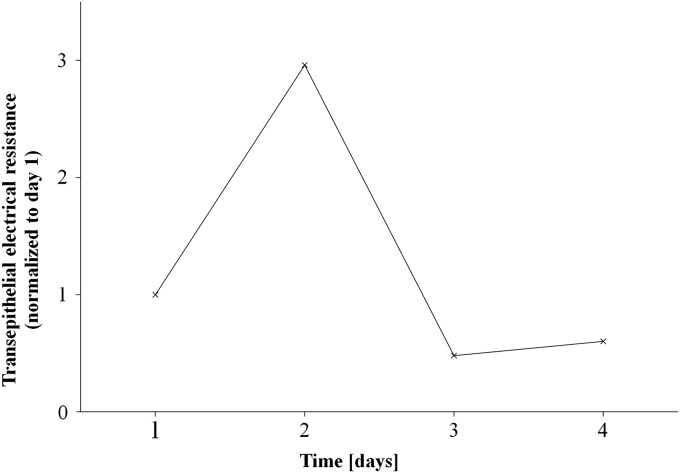
Representative transepithelial electrical resistance (TEER) curve of sprayed cells in inserts for days 1–4.

For long-term culture of RECs, the cells were directly sprayed into Transwell^®^ inserts (Corning) in a 12 well-plate with the same density as described earlier with *n*=3. The insert membrane was precoated with collagen (from human placenta, Bornstein and Traub Type IV, 0.7 μg/cm^2^; Sigma-Aldrich). Transepithelial electrical resistance was measured (see Fig. 2 for representative curve). After 7 days of culture, air–liquid interface culture was established and a modified Airway Epithelial Cell Growth Medium with retinoic acid was used (50 nM, Sigma-Aldrich; protocol from PromoCell). The medium was changed every other day.

### Live-dead staining

For cell survival studies, live-dead staining was conducted with calcein AM (AAT Bioquest^®^) and propidium iodide (PI; Sigma-Aldrich). First, cells were detached from the cell culture flasks, counted with CASY^®^ cell counter (Schärfe Systems), and resuspended in PBS or medium. Then, living cells were stained with calcein AM (4 μM) for 30–60 min at 37°C. After performing the spraying experiment, PI (1 μg/mL) was added to stain dead cells. Images were acquired directly with a fluorescence microscope (Observer Z1 and Axio Imager; Carl Zeiss) and a high-resolution CCD camera (AxioCam MRC; Carl Zeiss).

### Image processing

For automated analysis, the images were processed with a self-written macro for counting particles in ImageJ (Version 1.44p). All images were processed with the same parameters to ensure comparability.

### XTT assay

To analyze whether spraying influences the proliferation, an XTT assay (Roche Diagnostics) with *n*=5 was accomplished. Cells were sprayed in a 6-well plate (CellStar; Greiner Bio-One), and the suspension was then transferred into a 96-well plate (CellStar; Greiner Bio-One) for measuring. As a positive control, nonsprayed cells were pipetted into the wells; as a negative control, we used DMEM without fetal calf serum supplementation. On day 1, 3, and 6 after seeding, an XTT-reagent/coupling solution mixture (50:1) was added to each well; absorption was measured at 475 nm after 1 h of incubation at 37°C.

### Lactate dehydrogenase measurements

For measuring the lactate dehydrogenase (LDH) level in the cell culture medium, cells were sprayed or pipetted in a 24-well plate at the density noted earlier. On day 1, 3, and 6, the medium was changed and LDH level was determined by our laboratory service. As a positive control, the well plate was put in a −80°C freezer for 30 min.

### Histology and immunohistochemistry

The cells of the short-term experiment were fixed in the well plates with 4% formalin in PBS for 5 min and then rinsed with PBS. Nonspecific blocking and permeabilization was conducted by incubation with 5% normal goat serum (NGS; Dako) in 0.01% triton (Sigma-Aldrich) for 30 min. Primary antibodies were incubated for 1 h at 37°C. After rinsing the wells with PBS, the secondary antibodies were incubated for 30 min at 37°C. The cells were rinsed with PBS and counterstained with DAPI (Carl Roth^®^) for 5 min. After three washing steps, the plate was viewed with the fluorescence microscope and images were acquired with a high-resolution CCD camera.

For vSMCs, the first antibody was anti-α-smooth muscle actin (species: mouse; Sigma-Aldrich) with a concentration of 1:1000 in 5% NGS in 0.01% triton. As a secondary antibody, Alexa Fluor-594 (goat anti-mouse IgG; Life Technologies) was used with a concentration of 1:400. For RECs, as a first antibody we used anti-pan-cytokeratin (species: rabbit; Acris) with a concentration of 1:100. As a secondary antibody, Alexa Fluor-488 (goat anti-rabbit IgG; Life Technologies) was used with a concentration of 1:400. As a negative control, only the secondary antibody was used; exposure time was determined as the time that does not show any staining in the negative control. Preparation for histology, periodic acid Schiff's reaction (PAS), and pan-cytokeratin staining of paraffin sections was conducted as described earlier by Cornelissen et al.^19^

### Statistical analysis

Mean values and standard deviations were calculated for each parameter. Finally, statistical significance between controls and study groups was tested with an unpaired, two-tailed Student's *t*-test in Microsoft Office Professional Plus 2010 Excel Version 14. A *p*-value below 0.05 was considered significant.

## Results

### Cell survival depending on air pressure and cell suspension velocity

**FIG. 3.**
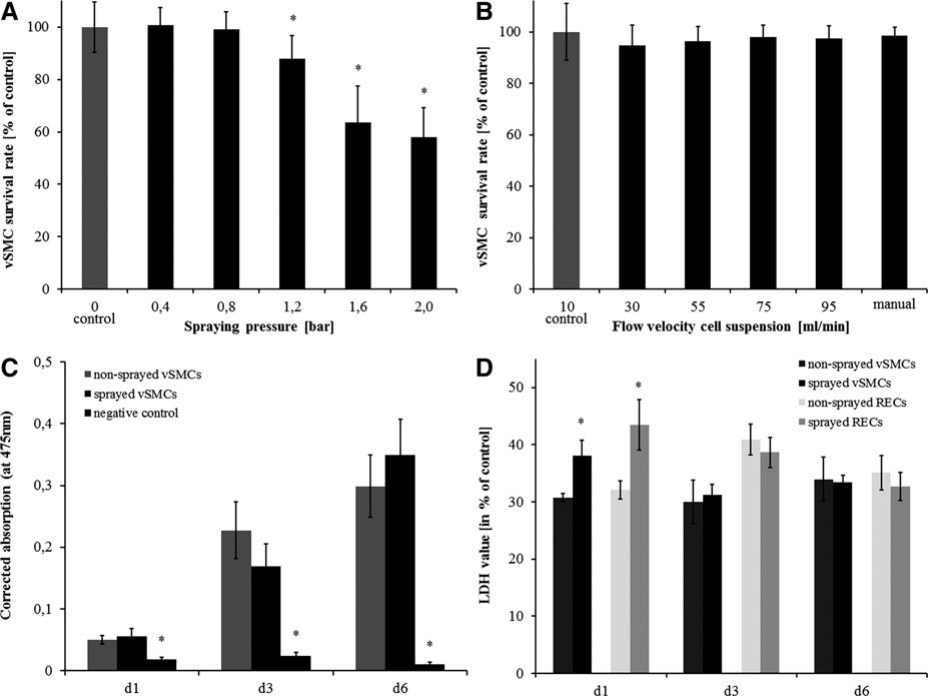
Influence of spraying on cells. **(A, B)** vSMC survival depending on spray parameters assessed directly after spraying with calcein AM/propidium iodide staining. **(A)** vSMC survival depending on spray pressures between 0 and 2 bar; cell survival is comparable to the control till a pressure of 0.8 bar. **(B)** vSMC survival depending on cell suspension velocities; there is no influence on cell survival by flow velocities. **(C)** XTT assay with vSMCs: There is no significant difference in proliferation after 1, 3, and 6 days. **(D)** LDH levels of the sprayed cells compared with nonsprayed controls in % of the positive controls. The LDH level of sprayed cells is significantly increased on day 1, but already normalized on day 3 (error bars indicate standard deviation, *p<0.05 to control). LDH, lactate dehydrogenase; vSMC, vascular smooth muscle cell.

To assess general suitability of the cell spraying process with the Tisseel EASYSPRAY set and to find the best parameters for the process, a first set of experiments was conducted with vSMCs. We tested the influence of air pressure and cell suspension velocity on cell survival directly after spraying with a calcein AM/PI-staining. As shown in Figure 3A, six different air pressures between 0 (as a control) and 2 bar were tested. For pressures of 0.4 and 0.8 bar, cell survival was 100.7% and 99.2% as high as the control. From a pressure of 1.2 bar, the survival decreased significantly: Cell survival was 87.9%, 63.7%, and 57.9% of the control for 1.2, 1.6, and 2 bar, respectively. Thus, spray pressure was adjusted to 0.4 bar for all the next experiments.

As another important parameter, we have tested the influence of the cell suspension velocity on cell survival. Other than the air pressure, the cell suspension velocity did not have an influence on cell survival. Values varied between 94.6% and 98.5% without any statistically significant difference (Fig. 3B). For further spraying experiments, cell suspension was injected slowly by hand (∼7–10 mL/min).

As RECs are, in general, more sensitive than vSMCs and isolation and culture is complex, we have tested the influence of the parameters determined earlier on the survival. In contrast to vSMCs, the survival decreased significantly: The survival rate of RECs when spraying with 0.4 bar was 88.5%. The survival rate might be simply compensated by increasing the amount of the cells in the suspension by 13%.

### XTT and LDH

As shown in Figure 2C, there is no significant difference between sprayed cells and control by means of mitochondrial activity on days 1, 3, and 6 after the experiment. Thus, spraying does not significantly influence the proliferation of vSMCs.

To assess the influence of the spray process on apoptosis or necrosis, the level of the intracellular enzyme LDH in the cell culture medium was examined on days 1, 3, and 6 after spraying for vSMC and RECs (Fig. 3D). On day 1, LDH level was significantly increased in the sprayed samples for both cell types. On days 3 and 6, no increased LDH level could be detected and there was no significant difference between sprayed samples and controls anymore.

### Differentiation of vSMCs and RECs

**FIG. 4.**
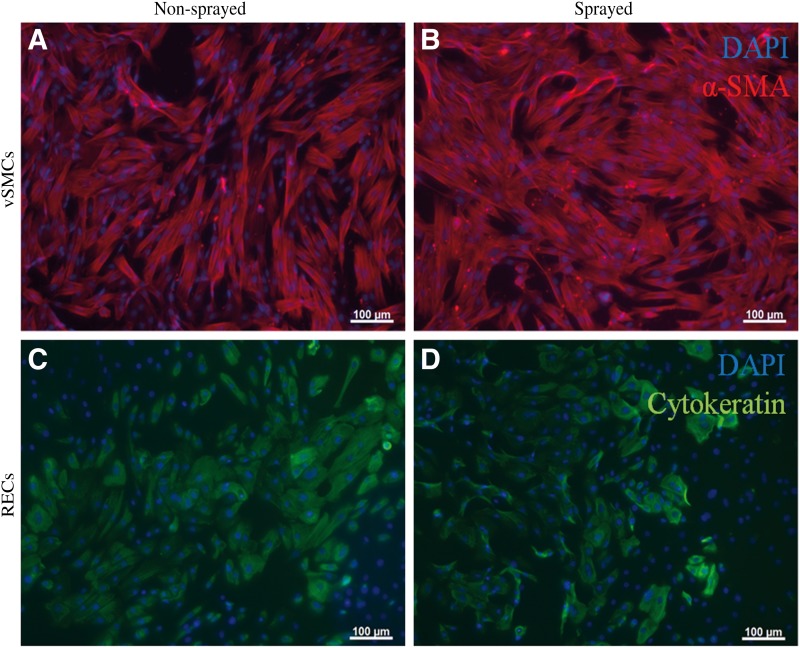
Ability of vSMCs **(A, B)** and RECs to differentiate **(C, D)** after spray processing with 0.4 bar. **(A, B)** vSMCs were stained with α-SMA antibody after 5 days of culture. Expression of α-SMA is similar in control and sprayed cells. **(C, D)** RECs were stained with pan-cytokeratin antibody after 3 days of culture. Expression of cytokeratins is similar in control and sprayed cells. Scale bars: 100 μm. α-SMA, α-smooth muscle actin; RECs, respiratory epithelial cells.

For proving cell differentiation potential in the short term after spraying, an immunohistochemical staining was conducted with antibodies against α-smooth muscle actin (α-SMA) and pan-cytokeratin for vSMCs and RECs, respectively. The results are shown in Figure 4. In the upper row (Fig. 4A, B), images for vSMCs are depicted. When comparing the nonsprayed and sprayed cells, there is no difference in staining or intensity according to visual judgment of the microscopic images. All cells are stained with α-SMA. Similar results are given for RECs in Figure 4C and D. Here, the majority of the cells are stained in both assays.

### Long-term culture of RECs without and with fibrin gel

As RECs are a very sensitive cell type and dedifferentiate quickly *in vitro*, we have tested differentiation and ciliary development in long-term culture after spraying. Here, an experimental group was added, in which the cells were sprayed within a fibrin gel, as this is particularly interesting for tissue engineering approaches. Embedding the cells in fibrin gel will help the cells stay in place when applied onto uneven surfaces or on the inside of tubular constructs.

**FIG. 5.**
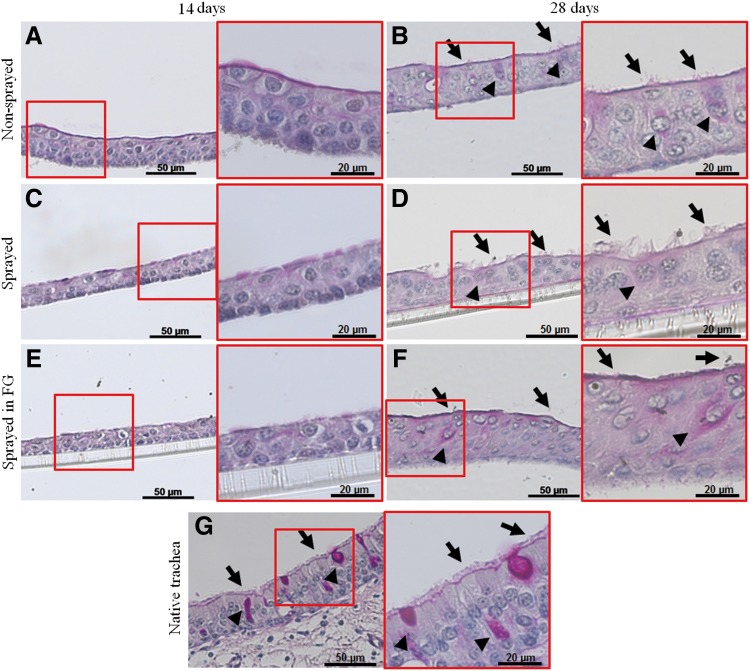
PAS staining of long-term culture air-liquid interface of RECs after spraying with and without fibrin gel after 14 and 28 days. **(A, B)** Nonsprayed control, **(C, D)** sprayed without FG, **(E, F)** sprayed with fibrin gel, **(G)** native trachea. After 14 days, RECs build up a layered structure with recognizable basal and apical cells in all assays. After 28 days, cilia are visible on the apical surface (arrows) and goblet cells are formed (arrowheads). In the cells sprayed within fibrin gel, fewer cilia are seen. Air-liquid interface was achieved by culture in insets. Scale bars: 50 μm in overview, 20 μm in magnification. FG, fibrin gel; PAS, periodic acid Schiff's reaction.

**FIG. 6.**
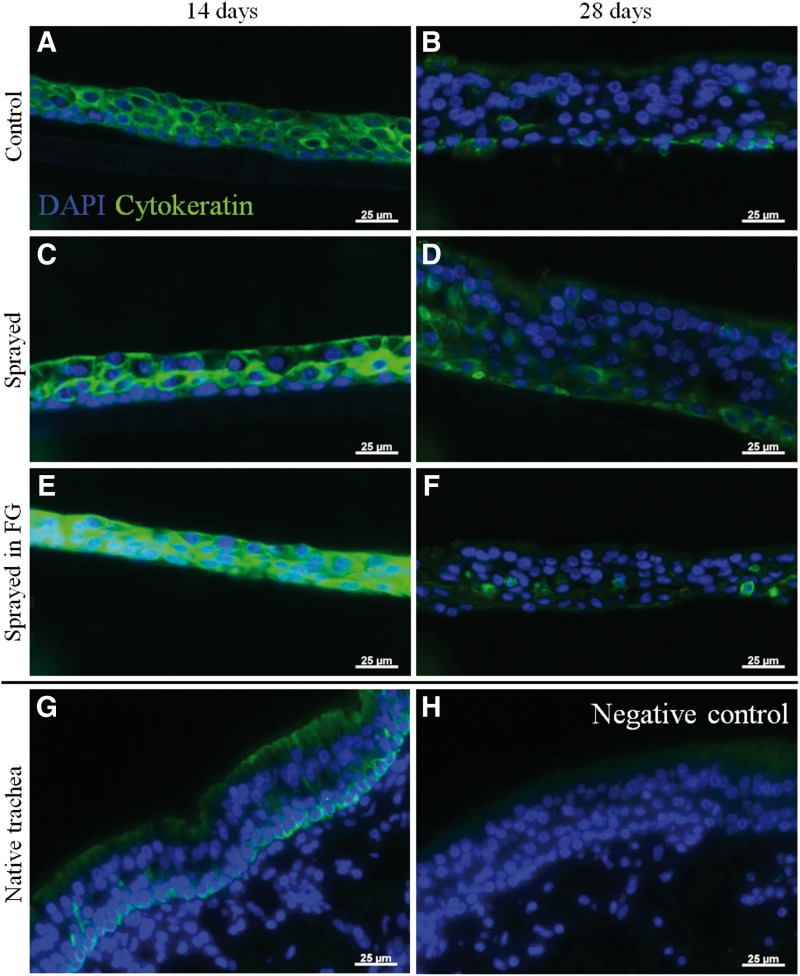
Immunohistochemistry against pan-cytokeratin of long-term culture of RECs after spraying with and without fibrin gel at day 14 and 28. **(A, B)** Nonsprayed control, **(C, D)** sprayed without fibrin gel, **(E, F)** sprayed with fibrin gel, **(G)** native trachea, **(H)** negative control. At day 14, all samples show a uniform distribution of fluorescent staining, whereas after 28 days and in the native tissue the basal cells have a higher level than the apical cells. Negative control: no primary antibody. Scale bars: 25 μm.

The results are shown in Figures 5 and 6. Histological images stained with PAS are shown for nonsprayed cells (Fig. 5A, B), sprayed cells (Fig. 5C, D), sprayed cells within fibrin gel (Fig. 5E, F), and native ovine trachea (Fig. 5G). After 14 days, RECs have built up a confluent epithelial layer with a pseudostratified appearance. In all assays, there is a layer of basal cells that have a smaller nucleus-to-cytosol ratio. After 28 days, in the sprayed and nonsprayed assay, the apical cells build up a mostly ciliated surface that is comparable to the native tracheal epithelium (Fig. 5, arrows). At that time point, there are also goblet cells in all samples (Fig. 5, arrowheads). However, these are not as distinct as in the native tissue. When spraying cells in fibrin gel, the ciliary development is not as advanced as in the other assays.

The immunohistochemical staining against pan-cytokeratin (Fig. 6) demonstrates a strong staining for all epithelial cells of the cultured constructs after 14 days. Thus, no dedifferentiation has taken place. In all experimental samples, the expression of cytokeratins is more uniform than in the native tissue. After 28 days, basal cells show a stronger staining than apical ones that is comparable to the expression pattern of the native tissue.

Thus, we show here that spraying of RECs without and with fibrin gel with an air pressure of 0.4 bar does not alter cell morphology, differentiation, ciliary development, and pan-cytokeratin expression pattern.

## Discussion

We proposed spray application of ovine vSMCs and RECs for tissue engineering of tubular constructs. As particularly RECs are sensitive and their differentiation *in vitro* is a challenge, a positive outcome of this study was uncertain. The study of Roberts et al. had shown first promising results for ovine RECs in terms of growth kinetics but not shown results for survival rates or with regard to epithelial differentiation.^6^ Here, we were able to show epithelial differentiation by means of pan-cytokeratin expression and ciliary development of ovine RECs with no evident long-term influence of the spray application.

In the literature, several methods and protocols can be found for spraying of various different cell types. One important objective for spraying experiments was given by Veazey et al., who proposed to reach fibroblast survival rates of more than 50% as the goal of their study.^4^ This objective was exceeded by far in our study. To find optimal conditions by means of pressure and velocity, vSMCs were analyzed. We did not see any change in survival rates compared with the control for pressures till 0.8 bar. Similarly, the XTT assay has not shown any significant difference in proliferation. The increased LDH level for both cell types on day 1, however, shows that the spraying puts the cells under stress for a very limited time, as it is already normalized on day 3. Thus, spraying does not have a long-term effect on survival or necrosis and cells do not respond negatively to the applied shear and elongation stresses. Similar results were reported by Schlabe et al. for dermal papilla and dermal sheath cells: The LDH level was higher only within the first medium change, whereas the growth rate was not altered.^23^ Further effects of spraying on the cells were excluded by immunohistochemistry and histology, where the sprayed and nonsprayed cells show comparable results. However, epithelial cells sprayed in a thin layer of fibrin gel show less cilia on their surface. This might be because the fibrin gel slows differentiation of the cells until it is degraded, thus deferring the differentiation by some days, though this was not proved.

Several other groups have assessed the survival rates of cells after spraying with different air pressures with airbrush devices. Spraying with higher pressures can produce a different spray, resulting in finer particles or a more uniform cell layer. Still, this is highly dependent on the spraying device used. With our setup, a good spray was already produced with a pressure of 0.4 bar. In general, next to the nozzle dimension, the pressure is a process parameter that is related to the shear the cells may experience during spray formation. Veazey et al. tested air pressures between 0.41 and 1.24 bar and showed a direct dependence of the survival of bovine dermal fibroblasts.^4^ Similarly, Nahmias et al. report survival rates of NIH 3T3 cells of 64% at a pressure of 0.97 bar till 90% for a pressure less than 0.34 bar.^3^ Tritz et al. have shown chondrocyte survival rates of 88% and 80% in alginate gels 3 days after spraying for pressures of 0.9 and 1.2 bar.^2^ In another study, the same group reports a survival rate of 52% for human mesenchymal stem cells in the same setup with a pressure of 0.9 bar.^24^ Roberts et al. report a chondrocyte survival of 70–84% depending on air flow rates between 4 and 8 L/min (which corresponds to different air pressures as well).^6^ Thus, higher pressures evoke higher shear and elongation stresses on the cells and reduce their survival. Hence, the unchanged survival of vSMCs and 88.5% survival rate of our RECs at a pressure of 0.4 bar are very good results.

Duncan et al. propose that survival studies after spraying alone do not suffice to validate the influence of spraying on cells.^10^ We could exclude an effect on cell lysis with normal LDH-levels in the cell culture medium from day 3. Furthermore, we assessed expression of α-SMA and pan-cytokeratin for vSMCs and RECs, respectively, after 3–5 days in culture and demonstrated an expression pattern comparable to the control. RECs were also cultured till 28 days and showed enhanced differentiation with goblet cell appearance, columnar shape of the apical cells, and pan-cytokeratin expression comparable to the native control. In both assays without fibrin gel, most cells were ciliated after this period, and cells sprayed with fibrin gel show fewer and less developed cilia probably due to initial fibrin gel degradation.

Thus, spraying is a highly promising procedure for application of RECs. As mentioned earlier, this is of special significance for tissue engineering approaches to develop functional constructs for trachea or bronchi. Experiments in this specific field have not yet been conducted. However, with the approach of embedding the RECs in fibrin gel, one can imagine spray coating even difficult topographies such as tubes. This is of particular interest, as fibrin gel was already proved to be a suitable scaffold for RECs.^19^ In literature, various approaches are reported where fibrin gel is used as a carrier for application of cells. Most of these have shown successful application of keratinocytes onto wounds.^7,13,14,25^ Still, some studies were focused on the development of tissue-engineered constructs: The team of Steinhoff has spray-applied stem cells or stem cell/endothelial cell mixtures in a fibrin glue for coating of heart valve scaffolds and report the desired homogeneous cell distribution.^8,26^ Farhat et al. were already able to demonstrate tissue engineering of porcine urothelium successfully *in vivo.*^15^ In addition to better cell adhesion, fibrin supports cell growth by enhanced diffusion of growth factors and by acting as a nutrient. Furthermore, it can be produced autologously and is biodegradable. Thus, spray application of cells in fibrin gel can be seen as a highly viable and promising option for tissue engineering approaches.

In this study, the influence of air pressures and cell suspension velocity on cell survival and differentiation capacity was tested to determine optimal spraying parameters without adverse effects. Apart from a slight reduction of the survival rate in RECs, no evident influence of spraying was found in longer culture periods. However, the embedding of cells in fibrin gel seems to at least slow down the ciliary development.

Spraying of vSMCs and RECs is a highly promising approach for coating of tissue-engineered constructs. When spraying the cells with fibrin gel, unfavorable topographies (as the lumen of tubular constructs) can be coated evenly with a thin cell layer. In future studies, we will assess the luminal coating with cells in detail.

## Abbreviations Used

α-SMA: α-smooth muscle actin
DMEM: Dulbecco's modified essential medium
LDH: lactate dehydrogenase
NGS: normal goat serum
PAS: periodic acid Schiff's reaction
PBS: phosphate-buffered saline
PI: propidium iodide
RECs: respiratory epithelial cells
vSMCs: vascular smooth muscle cells
XTT: 2,3-bis(2-methoxy-4-nitro-5-sulfophenyl)-2H-tetrazolium-5-carboxanilide
